# Pre-hospital ECPR in an Australian metropolitan setting: a single-arm feasibility assessment—The CPR, pre-hospital ECPR and early reperfusion (CHEER3) study

**DOI:** 10.1186/s13049-023-01163-0

**Published:** 2023-12-13

**Authors:** S. A. C. Richardson, D. Anderson, A. J. C. Burrell, T. Byrne, J. Coull, A. Diehl, D. Gantner, K. Hoffman, A. Hooper, S. Hopkins, J. Ihle, P. Joyce, M. Le Guen, E. Mahony, S. McGloughlin, Z. Nehme, C. P. Nickson, P. Nixon, J. Orosz, B. Riley, J. Sheldrake, D. Stub, M. Thornton, A. Udy, V. Pellegrino, S. Bernard

**Affiliations:** 1 The Alfred Hospital, Melbourne, Australia; 2 Ambulance Victoria, Melbourne, Australia; 3 Department of Public Health and Preventive Medicine, Monash University, Melbourne, Australia; 4 Department of Paramedicine, Monash University, Melbourne, Australia

## Abstract

**Introduction:**

Survival from refractory out of hospital cardiac arrest (OHCA) without timely return of spontaneous circulation (ROSC) utilising conventional advanced cardiac life support (ACLS) therapies is dismal. CHEER3 was a safety and feasibility study of pre-hospital deployed extracorporeal membrane oxygenation (ECMO) during cardiopulmonary resuscitation (ECPR) for refractory OHCA in metropolitan Australia.

**Methods:**

This was a single jurisdiction, single-arm feasibility study. Physicians, with pre-existing ECMO expertise, responded to witnessed OHCA, age < 65 yrs, within 30 min driving-time, using an ECMO equipped rapid response vehicle. If pre-hospital ECPR was undertaken, patients were transported to hospital for investigations and therapies including emergent coronary catheterisation, and standard intensive care (ICU) therapy until either cardiac and neurological recovery or palliation occurred. Analyses were descriptive.

**Results:**

From February 2020 to May 2023, over 117 days, the team responded to 709 “potential cardiac arrest” emergency calls. 358 were confirmed OHCA. Time from emergency call to scene arrival was 27 min (15–37 min). 10 patients fulfilled the pre-defined inclusion criteria and all were successfully cannulated on scene. Time from emergency call to ECMO initiation was 50 min (35–62 min). Time from decision to ECMO support was 16 min (11–26 min). CPR duration was 46 min (32–62 min). All 10 patients were transferred to hospital for investigations and therapy. 4 patients (40%) survived to hospital discharge neurologically intact (CPC 1/2).

**Conclusion:**

Pre-hospital ECPR was feasible, using an experienced ECMO team from a single-centre. Overall survival was promising in this highly selected group. Further prospective studies are now warranted.

## Introduction

The current treatment of out of hospital cardiac arrest (OHCA) in metropolitan Melbourne (population: 4.9 million, area: 9900 km^2^) is conventional cardiopulmonary resuscitation (CPR) and advanced cardiac life support (ACLS) by ambulance paramedics and includes chest compressions, ventilation with oxygen, defibrillation and intravenous administration of medications (epinephrine and amiodarone). In about half OHCA cases [1], where resuscitation is attempted, paramedics do not achieve return of spontaneous circulation (ROSC) and, after 45 min, the patient may be declared deceased at the scene as per Ambulance Victoria Clinical Practice Guidelines (CPGs) [2].

If ROSC is not achieved after 30 min of advanced life support, expected ROSC may be as low as 2–6% [3], with neurologically intact survival of less than 1% [4, 5]. In 2020/21 in Victoria, less than 3% of cardiac arrests were transported to hospital with CPR ongoing that may have been considered for Extracorporeal Membrane Oxygenation (ECMO) CPR (ECPR) [1]. The use of ECPR for selected cases of refractory OHCA is now supported by various international guidelines [6–8].

In a trial of emergency department (ED) initiated ECPR for OHCA, five of eleven (45%) patients in the OHCA cohort survived in our original CHEER trial [9]. However, between 2016 and 2019 in metropolitan Melbourne, just 49 OHCA patients were transported with CPR ongoing to an ECPR capable hospital for consideration of ED initiated ECPR. 23 underwent ECPR, with 4/23 (17%) neurologically intact survivors, cerebral performance category (CPC) 1 or 2 [10]. In those who did not receive ECPR, 1 out of 26 (4%) survived neurologically intact. This gave an overall survival of 5/49 (10.2%) for those transported with ongoing mechanical CPR without ROSC. Time from emergency call to hospital arrival was 64 min (IQR 54–82 min) resulting in many patients being declined ECPR due to excessive CPR “low flow” times. Despite the low transportation rate with CPR ongoing, the region’s overall survival from OHCA for the “Utstein” [11] patient group was 39% over this time period [1], indicating a well functioning cardiac arrest response system.

Pre-hospital ECPR has been tested in other metropolitan jurisdictions including Paris, France, with published survival rates of 8–29% [12], with a reported time from cardiac arrest to establishment of ECMO support of 87 min (± 27 min).

The CHEER3 trial was designed as a single-arm feasibility and safety trial of pre-hospital ECPR in the Melbourne metropolitan area. Our primary hypothesis was that pre-hospital ECPR could be delivered safely and quickly in this setting, with favourable times to initiation of veno-arterial (V-A) ECMO compared to historical hospital-based ECPR for OHCA utilising an ultrasound guided percutaneous technique.

## Methods

### Study design

The study was an investigator-initiated, prospective feasibility study involving the provision of ECPR in the prehospital setting. Approval to undertake the study was granted by The Alfred Hospital Human Research Ethics Committee (HREC Project Number: 53/19), with a waiver of individual patient informed consent, given the emergent nature of the study intervention.

### Disclosures

Support for the study was provided by a collaboration between Ambulance Victoria and The Alfred Hospital for staff hours, vehicle provision and equipment. Financial support was provided by a donation from The Alfred Foundation (grant number IPAP2018/0136).

### Study population

Table 1.

**Table 1:** Patient eligibility

Inclusion Criteria:	Exclusion Criteria:
18–65 years old	< 20 min conventional ACLS therapies
Witnessed arrest	Previously known life limiting co-morbidities (e.g. end stage heart failure / COPD) or terminal illness
Initial cardiac rhythm VF/VT/PEA	ROSC with sustained haemodynamic recovery
Time to commence CPR < 5 min (“no-flow”) by bystander or EMS	Pre-arrest cerebral performance score (CPC) 3 or 4
Time to commence cannulation < 45 min (“low-flow”) from arrest (unless periods of ROSC or “signs of life” during resuscitation	Femoral cannulation anatomically impossible (eg iliofemoral occlusion)

*CPR* Cardiopulmonary Resuscitation, *EMS* Emergency Medical Service, *ROSC* Return of spontaneous circulation, *VF*ventricular fibrillation, *VT* ventricular tachycardia, *PEA* pulseless electrical activity, *ACLS* Advanced Cardiac Life Support, *COPD* Chronic Obstructive Pulmonary Disease

### Intervention

Resuscitation care by the emergency medical service (EMS), Ambulance Victoria, was initiated as per their standard guidelines [2]. This included high-performance CPR, defibrillation, intravenous or intraosseous access, medication administration, and assisted ventilation via supraglottic airway or endotracheal tube [13]. A minimum of 20 min of conventional CPR and ACLS was performed prior to consideration of ECPR.

In eligible cases, a three-person team consisting of two intensive care consultant physicians, trained and accredited in ultrasound guided femoral cannulation and ECMO deployment, and an experienced Intensive Care Paramedic (ICP) trained in ECMO circuit priming and pump management were dispatched from an inner metropolitan Melbourne quaternary hospital.

Suspected OHCA identified in the “000” EMS call were allocated to the ECMO response team automatically. The ICP secondarily screened incoming data about these calls and, if thought likely to fulfil inclusion criteria, activated a response by the ECMO team. Utilising the situational report of the first arriving paramedic crew, and with the information provided in subsequent situational reports, the ECMO team either cancelled or continued to the scene depending on the presence of exclusion criteria.

Eligibility criteria were assessed on arrival of the ECMO team by the lead physician, if all inclusion criteria were met the decision to initiate ECPR (“decision time”) was conveyed to all attending emergency personnel. At this point the team prepared the patient and surroundings for cannulation and initiation of V-A ECMO support via a femoro-femoral cannulation. The patient was intubated with an endotracheal tube, if not already done, by the treating paramedic team and mechanical CPR (Corpuls) commenced, if not already applied. The patient’s femoral regions were exposed, betadine applied and a sterile field created. A 19Fr multistage venous access cannula (Gettinge) was introduced using an ultrasound guided (Butterfly inc) Seldinger percutaneous technique into the femoral vein, with the tip advanced to the right atrium—superior vena cava junction. A 15Fr arterial return cannula (Gettinge) was placed in the common femoral artery utilising an ultrasound guided Seldinger percutaneous technique with the tip in the distal abdominal aorta or proximal iliac artery. These cannulae were connected to a portable ECMO pump (CardioHelp, Gettinge) using a circuit (HLS, Gettinge) and whole body perfusion at a minimum of 3.0L/minute blood flow was commenced. Chest compressions were stopped once V-A ECMO blood flow was greater than 3.0L/min; this was defined as the time of ECMO support commencement.

Further attempts at defibrillation to achieve some native ROSC were attempted 5–10 min after establishment of support if a shockable rhythm persisted. 2 g of IV cefazolin was administered at the scene if there was no known allergy to cephalosporins.

**Fig. 1:**
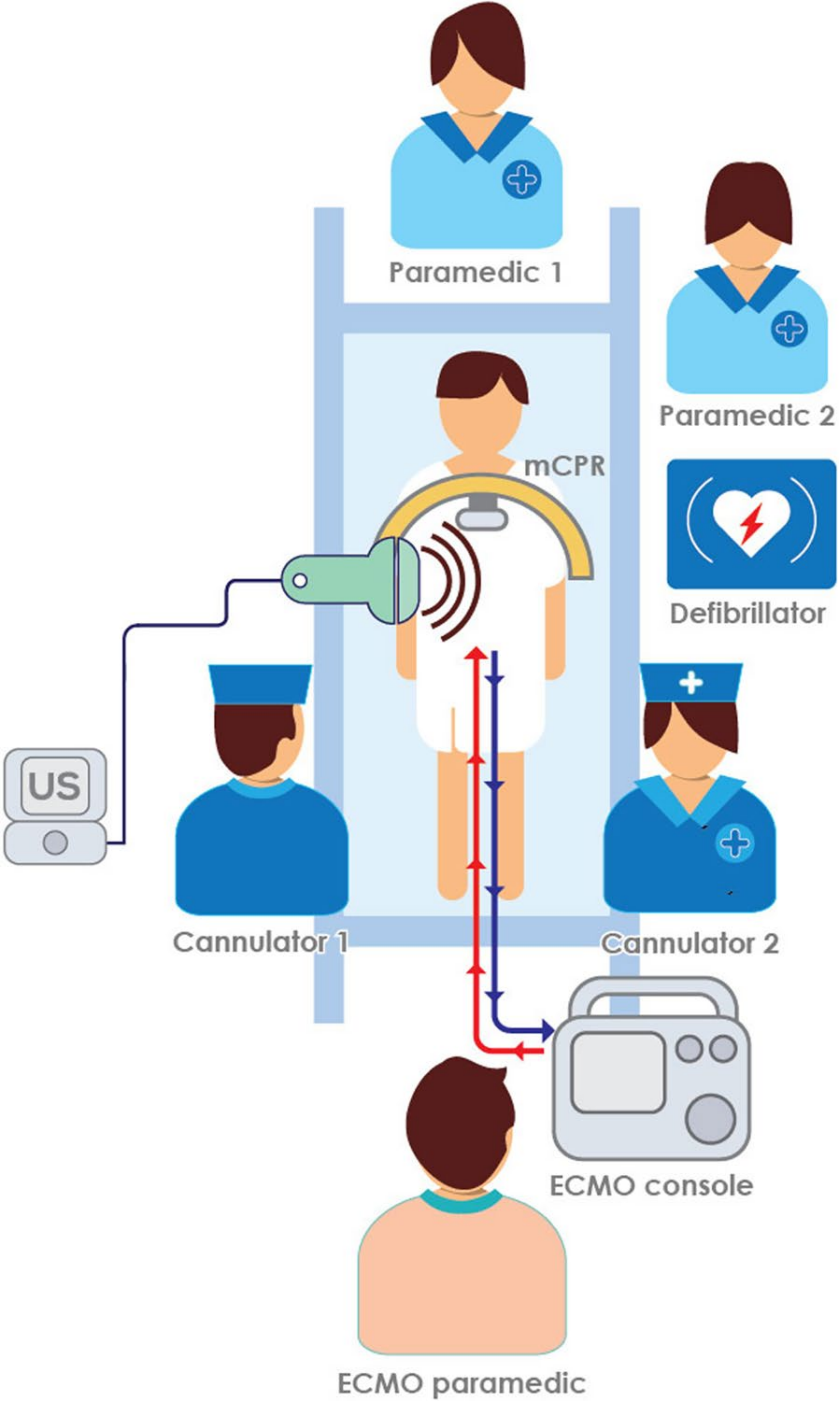
Pre-hospital ECPR cannulation setup

The patient was then extricated from the scene, pre-hospital notification to the coronary catheterisation laboratory given, and the patient was transported to the Emergency Department for ongoing investigations and management. Post resuscitation care occurred in a quaternary intensive care unit (ICU) with an established ECMO program, and was standardised as per local OHCA / ECPR guidelines. This included early coronary angiography ± percutaneous coronary intervention (PCI), targeted temperature management (TTM) at 36.0 C for 24 h, and routine placement of a 6 Fr distal perfusion cannula (Arrow) into the superficial femoral artery on the side of arterial ECMO cannulation to reduce the risk of limb ischaemia. Neurological assessment was conducted from 108 h post cardiac arrest if the patient could be supported to that time point, or earlier if progression to brain death had occurred (Fig. 1).

## Results

**Fig. 2:**
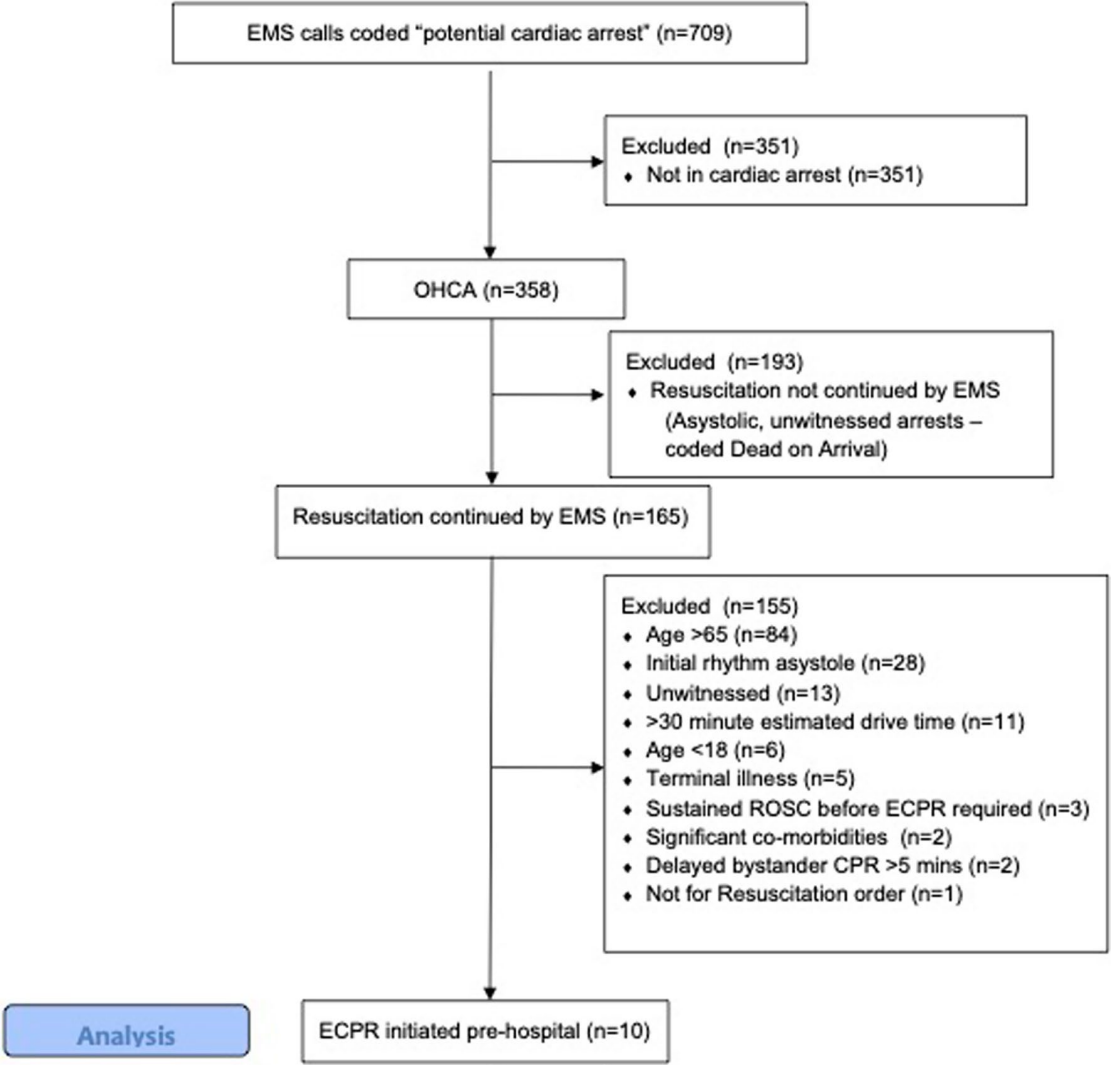
Dispatch data

From February 2020 to May 2023, the trial recruited two days per week during daytime hours (0830–1700), interspersed with periods when recruitment was ceased due to the competing critical care demands of the COVID pandemic. The total period of recruitment spanned 117 days, over which the ECMO team responded to 709 dispatches coded as potential cardiac arrests of medical cause. A breakdown of these are provided in Fig. 2, with the number of exclusion criteria met.

Of 709 EMS calls coded as potential cardiac arrest, 358 were confirmed cardiac arrests. Resuscitation was continued by EMS in 165 cases, of which 155 were excluded. 10 fulfilled all inclusion criteria. All 10 (100%) were successfully cannulated and established on ECPR at the scene (Fig. 2).

All patients recruited were men with average age 46 (20–62) years with no known pre-existing ischaemic heart disease. Ventricular Fibrillation (VF) was the most common presenting cardiac rhythm.

The ECPR team was deployed from the hospital and arrived at the scene of the cardiac arrest in 27 min (range 9–37 min) from the time of initial EMS call. Mean time from pre-hospital decision to initiate ECPR to establishment of whole body V-A ECMO perfusion at > 3L/min was 16 min (range 11–26 min). Nine cannulations were performed on the ground and one was performed on a stretcher after a patient was relocated from a highly public area.

Three of the patients had a period of intermittent ROSC. Total ROSC time averaged 6 min (range 1—15 min) prior to re-arrest. This is why total CPR time was less than the time from emergency call to establishment of ECMO blood flow for some patients.

Seven of ten patients had proximal coronary lesions amenable to PCI. Two of ten patients had devastating Stanford type-A aortic dissections that were not amenable to operative management and were palliated within hours of arrival to hospital. One patient had a chronically occluded RCA that was not amenable to PCI.

Average length of ICU admission was 108 h (207 h for survivors and 41 h for non-survivors). Average length of hospital stay was 189 h (411 h for survivors, 41 h for non-survivors).

One patient became unsupportable within 24 h. Three patients developed severe hypoxic ischaemic encephalopathy (HIE) or brain death and were palliated between day 3 and day 5 of hospital admission.

Overall survival to hospital discharge was 4/10 (40%), all being neurologically intact, CPC 1 or 2. One patient’s neurological status improved from CPC 2 to CPC 1 at 6 months. One non-survivor became an organ and tissue donor after diagnosis of brain death (Tables 2, 3, 4).

**Table 2:** Demographics of patients fulfilling all inclusion criteria

Demographics	
n	10
Male, n, (%)	10 (100%)
Age mean (range), years	46 (20–62)
*Location of cardiac arrest*	
Work place n, (%)	5 (50%)
Public place n, (%)	4 (40%)
Home n, (%)	1 (10%)
Signs of Life during CPR, n, (%)	2 (20%)
Pupil size during CPR mean mm, (range)	4.6 (3–5)
CPR during ECMO cannulation n, (%)	10 (100%)
*Initial Rhythm*	
VF n, (%)	8 (80%)
VT (pulseless) n, (%)	1 (10%)
PEA n, (%)	1 (10%)
Bystander applied AED, n, (%)	5 (50%)
Defibrillation attempts if shockable rhythm, mean (range)	7 (4–12)
Epinephrine dosing intra-arrest, mean (range), mg	6 (3–8)
Amiodarone dosage intra-arrest, mean (range), mg (n = 9)	450 (450–450)
Intermittent ROSC during arrest n, (%)	3 (30%)
If intermittent ROSC—total duration of ROSC (range), mins	6 (1–15)
End tidal CO2 during arrest, mean (range), mmHg	32 (13–45)
Mechanical CPR device intra-arrest, n, (%)	8 (80%)
*Past Medical History*	
Known Ischaemic Heart Disease n, (%)	0 (0%)
Diabetes Mellitus n, (%)	1 (10%)
Smoking n, (%)	2 (20%)
Peripheral Vascular Disease n, (%)	0 (0%)
Cerebral Vascular Disease n, (%)	0 (0%)
Working prior to arrest n, (%)	10 (100%)

CPR; Cardiopulmonary Resuscitation, VF; Ventricular Fibrillation, VT; Ventricular Tachycardia, PEA; Pulseless Electrical Activity, AED; Automatic External Defibrillator

**Table 3:** Response and cannulation times / CPR durations / Initial ECMO parameters

ECPR details	
n	10
Total CPR duration, mean (range), min	46 (32—62)
EMS call to ECMO team arrival on scene, mean (range), min	27 (9—37)
Decision to ECMO blood flow > 3 Ltr/min, mean (range), min	16 (11—26)
EMS call to ECMO blood flow > 3 Ltr/min, mean (range), min	50 (35–62)
ECMO blood flow @ 5 min, mean (range), Ltr/min	3.2 (2.8–4.0)
Fresh Gas Flow @ 5 min, mean (range), Ltr/min	4.1 (4.0–5.0)
ECMO return pressure @ 5 min, mean (range), mmHg	274 (217–360)
ECMO access pressure @ 5 min, mean (range), mmHg	− 110 (− 80 to − 180)
Venous blood temperature @ 5 min, mean (range), celcius	34.9 (34.1–37.5)
*Native Rhythm on commencement of ECMO*	
PEA, n (%)	4
VF, n (%)	5
Asystole, (%)	1
Lactate @ hospital arrival, median (range),mmol/L	7.3 (1.7–16.2)
Troponin @ hospital arrival, median (range) ng/L	7,453 (118–98,000)
*Initial investigation*	
Coronary Angiography, n (%)	8
CT-angiography, n (%)	2
*Aetiology of arrest:*	
Proximal Left Anterior Descending / Left Circumflex lesion, n (%)	4
Left Main Stem lesion n (%)	2
Right coronary lesion, n (%)	1
Aortic Dissection, n (%)	2
No cause identified, n (%)	1
Percutaneous Coronary Intervention (PCI), n (%)	7

**Table 4:** Hospital data

ICU and Hospital care	
n	10
ICU Admission Length mean, (range) hours	108 (0.25–386)
ICU admission Length—*Survivors* mean, (range) hours	207 (138–386)
ICU admission—*Non-survivors* mean, (range) hours	41 (0.25–106)
Hospital Admission, mean, (range) hours	189 (0.25–576)
Hospital Admission—*Survivors* mean, (range) hours	411 (260–576)
Hospital Admission—*Non-survivors* mean, (range) hours	41 (0.25—106)
Renal Replacement Therapy	4 (40%)
Mechanical ventilation mean, (range) hours	90.75 (0.25–288)
Vasoactive medications mean, (range) hours	53.5 (0.25–120)
TTM target temperature 1st 24 h	35.9 (35.0–36.0)
*Hospital outcome*	
CPC 5 (Death)	6 (60%)
CPC 2	1 (10%)
CPC 1	3 (30%)
*6 month outcome*	
CPC 5 (Death)	6 (60%)
CPC 1	4 (40%)
Reasons for death or palliation:	n = 6
HIE / Brain Death	3/6 (50%)
Unsupportable circulation	1/6 (17%)
Inoperable pathology	2/6 (33%)
*ECMO related complications:*	
Cannulation complications	2 (20%)*
Failure to establish ECMO blood flow	0 (0%)
Bleeding complication (requiring transfusion)	1 (10%)**
Infection at cannulation site	0 (0%)
Limb ischaemia	0 (0%)
Mechanical device problem	0 (0%)
Organ and tissue donor after death	1/6 (17%)

*ICU* Intensive Care Unit, *TTM* Targeted Temperature Management, *CPC* Cerebral Performance Score, *HIE* Hypoxic Ischaemic Encephalopathy

^*^Unable to feed arterial guide wire, contralateral cannulation successful. No arterial back bleeding, contralateral cannulation successful

^**^Related to insertion of distal perfusion cannula (DPC) once in the ICU, required surgical exploration, repair and insertion of 6Fr DPC

## Discussion

### Key findings

This pilot study of patients with refractory OHCA demonstrated that pre-hospital, ultrasound guided ECPR was feasible, with 100% successful, rapid, scene-based establishment of V-A ECMO support. The application of pre-hospital ECPR resulted in a significant reduction in low-flow time, compared to historical data in our region, with a mean CPR duration of 46 min, and all patients being established on support within 62 min of the emergency call. Finally, none of the patients manifested significant adverse events related to prehospital cannulation, and overall in-hospital survival was encouraging at 40%.

### Relationship to previous literature

Based on the trial’s pre-defined inclusion criteria, ECPR appropriate arrests were rare; just 10 patients fulfilled all inclusion criteria out of 709 potential cardiac arrest EMS calls (1.4%). Approximately half of the EMS calls coded as potential arrest were found not to be in cardiac arrest. Of the 358 confirmed arrests, a further 193 did not receive ongoing resuscitation by EMS as per Ambulance Victoria Clinical Practice Guidelines, due to a prolonged unwitnessed period with asystole as presenting rhythm, where the benefits of ongoing resuscitation are considered futile. The 10 ECPR appropriate cases out of 358 confirmed arrests (2.8%) is consistent with other jurisdictions publishing data on ECPR for OHCA [14–17], and reflects that refractory, witnessed arrests in patients < 65 years of age constitute a minority of arrests seen in the general population.

This gave a utilisation rate of 1 prehospital ECPR for every 12 days the ECMO team was available to respond, 08:30–17:00, to a metropolitan population of circa 5 million. If the prevalence of appropriate arrests is evenly distributed over the week, this would equate to 30 appropriate patients per year for a 7 day per week, day-time capability.

Unwitnessed arrests, age over 65, and an initial rhythm of asystole were the largest causes of exclusion. Ongoing research into prompt identification of cardiac arrest and timely initiation of bystander CPR are needed to increase the pool of potentially salvageable cases. Similarly, the upper age limit employed in this study (65 years) was determined arbitrarily, and future work should utilise a data-driven cut-off, as determined by functional outcome and health economic analyses.

Time from EMS call to ECMO support, which has been demonstrated to have a significant impact on survival for OHCA salvaged with ECPR [17, 18], appeared promising with this ultrasound guided, prehospital deployed ECPR technique taking 50 min (35–62 min). This compares favourably to other published research from Paris: mean 87 min for prehospital ECPR [12], and for hospital based systems of ECPR for OHCA: Minnesota mean 59 minutes [15] and Prague mean 61 minutes [14]. It also compares favourably with our historical local data for OHCA transported to hospital for consideration of salvage ECPR with a mean time to hospital arrival of 64 min, excluding the time taken to initiate ECMO [10].

All the survivors had total CPR durations of less than 45 min and 3 of the 4 survivors had a period of intermittent ROSC during the arrest. This corroborates prior research that intermittent ROSC is a positive predictive factor for survival in ECPR [12, 14].

Hospital based delivery of salvage ECPR for refractory OHCA requires transporting patients with ongoing conventional CPR. Earlier transport to the hospital, to reduce low flow times, may compromise conventional CPR quality and potentially worsen outcomes. At present only a handful of OHCA are appropriate for salvage ECPR, therefore monitoring of outcomes for conventional CPR therapies is important, for example the Utstein group of OHCA [11]. This ensures changes to pre-hospital care to improve access to ECPR doesn't adversely affect outcomes for a larger group of patients who are currently ineligible to receive ECPR.

### Strengths and limitations

This study has a number of strengths. We believe we were able to identify all non-traumatic cardiac arrests within metropolitan Melbourne that had an emergency call initiated on the days and hours that the ECMO team were available, giving a valid sample of arrests within this jurisdiction who might be eligible for ECPR. Our inclusion criteria were similar to those of previously published ECPR series, improving the generalizability and comparability of our findings. Moreover, we followed all enrolled patients through to hospital discharge, providing robust data on clinical outcomes and processes of care.

We also acknowledge a number of limitations, including the highly selected nature of the study cohort. This was a single-jurisdiction, single-arm study, and the pre-hospital management of OHCA varies significantly between regions. As such, the applicability of ECPR in our study setting (metropolitan Melbourne, Australia) may not be universally applied to jurisdictions with differing EMS and pre-hospital systems. In addition, the lack of a randomised control arm limits any specific inference, albeit a number of process measures (time to support) and outcomes, were improved compared with historical data.

### Study implications

Our study implies that, in a highly selected cohort of refractory OHCA patients, it is feasible to deploy prehospital V-A ECMO as a salvage procedure. This has important ramifications for equity of access to this therapy.

Between Jan 2016 to Dec 2019, only 49 patients were transported to an ECPR centre in metropolitan Melbourne for consideration of salvage ECPR. Of these, only half had ECPR initiated due to excessive low flow times or other exclusion criteria, yielding an annual ECPR utilisation rate for OHCA of just 6 patients. Pre-hospital deployment of ECPR could potentially improve access to ECPR over a larger geographic area of metropolitan Melbourne (9900 Sq km) and increase utilisation to circa 30 cases per year, a fivefold increase for this highly selected group of OHCA patients. Moreover, we also anticipate that staffing a pre-hospital ECPR service “24/7”, would lead to a significant further increase in potential recipients.

At present the optimal model of ECPR initiation for OHCA is yet to be determined, be it hospital based, rendezvous or scene based delivery. Factors relating to local jurisdiction EMS capability, utilisation rates and resource availability are likely to influence which can achieve the best outcomes for patients.

## Conclusion

Pre-hospital deployment of ECPR for OHCA appeared to be feasible in this single jurisdiction, single-arm feasibility trial. Whilst neurologically intact survival appeared promising at 40%, further studies are warranted to investigate the optimum mode of delivery of ECPR in terms of efficacy and cost effectiveness for OHCA.

## Data Availability

Please contact author for data requests.
